# A prospective, randomised study to compare two palliative radiotherapy schedules for non-small-cell lung cancer (NSCLC)

**DOI:** 10.1038/sj.bjc.6602477

**Published:** 2005-03-15

**Authors:** E Senkus-Konefka, R Dziadziuszko, E Bednaruk-Młyński, A Pliszka, J Kubrak, A Lewandowska, K Małachowski, M Wierzchowski, M Matecka-Nowak, J Jassem

**Affiliations:** 1 Department of Oncology and Radiotherapy, Medical University of Gdańsk, Dębinki 7, 80-211 Gdańsk, Poland; 2 Pomeranian Medical Academy, Strzałowska 22, 71-730 Szczecin, Poland; 3 Oncology Center, Dr Izabeli Romanowskiej 2, 85-796 Bydgoszcz, Poland; 4 Lower Silesian Oncology Center, Hirszfelda 2, 53-413 Wrocław, Poland; 5 The Maria Skłodowska-Curie Memorial Cancer Center & Institute of Oncology, WK Roentgena 5, 02-781 Warszawa, Poland; 6 Greatpoland Cancer Centre, Garbary 15, 61-866 Poznań, Poland

**Keywords:** non-small-cell lung cancer, radiotherapy, palliative treatment

## Abstract

A prospective randomised study compared two palliative radiotherapy schedules for inoperable symptomatic non-small-cell lung cancer (NSCLC). After stratification, 100 patients were randomly assigned to 20 Gy/5 fractions (fr)/5 days (arm A) or 16 Gy/2 fr/day 1 and 8 (arm B). There were 90 men and 10 women aged 47–81 years (mean 66), performance status 1–4 (median 2). The major clinical characteristics and incidence and degree of initial disease-related symptoms were similar in both groups. Treatment effects were assessed using patient's chart, doctor's scoring of symptomatic change and chest X-ray. Study end points included degree and duration of symptomatic relief, treatment side effects, objective response rates and overall survival. A total of 55 patients were assigned to arm A and 45 to arm B. In all, 98 patients received assigned treatment, whereas two patients died before its termination. Treatment tolerance was good and did not differ between study arms. No significant differences between study arms were observed in the degree of relief of all analysed symptoms. Overall survival time differed significantly in favour of arm B (median 8.0 *vs* 5.3 months; *P*=0.016). Both irradiation schedules provided comparable, effective palliation of tumour-related symptoms. The improved overall survival and treatment convenience of 2-fraction schedule suggest its usefulness in the routine management of symptomatic inoperable NSCLC.

Lung cancer is the most common human malignancy worldwide, accounting for 1.2 million new cases and 1.1 million deaths a year (Parkin, 2001). Most patients present with inoperable tumour and the majority of disease symptoms are related to its local progression. In non-small-cell lung cancer (NSCLC) patients not suitable for surgery or radical (chemo)radiotherapy, the main aim of treatment is palliation. In these patients, palliative radiotherapy remains the main therapeutic modality. Given the short expected survival, treatment of these patients should be short and nondistressing (Durrant *et al*, 1971; Sundstrom *et al*, 2004). Over the last 30 years, several attempts have been made to develop treatment schedules combining effective symptom control and short treatment time. The benefits of such an approach include better comfort of patients having anyway short expected survival, and savings on the use of radiotherapy equipment, a resource still deficient in many countries. Additionally, shorter treatments generally allow hospitalisation to be avoided and enable earlier improvement of symptoms (Kowalska, 1992). The equivalence of shorter *vs* longer radiotherapy schemes in terms of symptom control was demonstrated in a series of randomised studies (Simpson *et al*, 1985; Medical Research Council Lung Cancer Working Party, 1991; Medical Research Council Lung Cancer Working Party, 1992; Abratt *et al*, 1995; Nestle *et al*, 2000; Kramer *et al*, 2003; Sundstrom *et al*, 2004). Nevertheless, doubts still exist regarding the potentially detrimental impact of shorter regimens on overall survival, particularly in patients with good performance status. In consequence, in many institutions this method has not been accepted as a standard of care. The aim of our study was to add to the evidence on the feasibility and equivalence of a 2-fraction (fr) *vs* commonly used 5-fraction regimen in terms of palliation of thoracic symptoms, toxicity and survival in the hope of optimising treatment practice in our country.

In the coordinating centre, hypofractionated radiotherapy in the palliative treatment of NSCLC was introduced in 1990. In a pilot study, a dose of 24 Gy in 3 fractions delivered on days 1, 8 and 22 was used (Drozd-Lula *et al*, 1996). The drawback of this regimen, however, was the long overall treatment time and concern about the relatively high dose to the spinal cord. Additionally, in most patients palliative effect was already observed after two fractions, and many were spared the third fraction. The last fraction was also abandoned in patients progressing after the first two fractions (Drozd-Lula *et al*, 1996). As a result, experience was gained with the dose of 16 Gy in 2 fractions 1 week apart, which was then chosen as the experimental arm for the current study. The control arm (20 Gy in 5 fractions over 5 consecutive days) was the regimen of palliative lung cancer irradiation most frequently used in Poland.

## MATERIALS AND METHODS

Eligibility criteria included cytologically or histopathologically confirmed NSCLC not suitable for radical treatment by surgery or radiotherapy, the presence of symptoms related to chest tumour (cough, dyspnoea, haemoptysis, chest pain, dysphagia or superior vena cava syndrome (SVCS)), age >18 years, WHO performance status ⩾1, expected survival of at least 3 months and written informed consent. Patients with both locally advanced and metastatic disease were allowed to participate. Ineligibility criteria included previous chest radiotherapy, systemic anticancer therapy given concurrently or planned within the next 6 weeks and difficulties expected with follow-up or with completing the ‘patient questionnaires’. Local ethics committee approval of the protocol was required.

Patients were randomised to receive 20 Gy in 5 fractions over 5 consecutive days (arm A) or 16 Gy in 2 fractions 1 week apart (arm B). Randomisation was conducted by means of a dedicated computer program, after stratification for treating centre, performance status (PS) and extent of disease.

Baseline examinations included history and physical examination, full blood count, chest X-ray and completion of the ‘patient questionnaire’. Doses of radiotherapy were prescribed to the mid-point without air correction, using two parallel-opposed megavoltage fields. No spinal cord shielding was used.

Primary end points included degree and duration of relief of chest tumour-related symptoms, as assessed by patients and physicians. Secondary end points were treatment side effects, objective response and overall survival. Assessed symptoms of chest tumour included cough, dyspnoea, haemoptysis, chest pain, dysphagia, SVCs or ‘other’ and were evaluated both by patients (at baseline, weekly for 8 weeks and then during follow-up visits) and by physicians (at baseline and during follow-up visits). The grading of symptom intensity was performed using a 4-point scale (none, mild, moderate, severe). The duration of improvement was expressed by the number of assessments (not necessarily consecutive) with symptomatic improvement recorded by the patient. Such a method was chosen to allow for variability of treatment effect over time. Physician assessment included also categorical estimation of the overall treatment effect (complete symptomatic response, improvement, no change or worsening of symptoms).

Follow-up visits were scheduled monthly for the first 6 months, bimonthly for the next 6 months and every 3 months thereafter. Chest X-rays were repeated bimonthly or when clinically indicated. In the case of poor response to radiotherapy or progression of symptoms, further treatment was left to the discretion of the treating physician.

Assuming a 50% improvement rate in the control arm (20 Gy/5 fr), to detect the difference in efficacy greater than 15% with a significance level of 0.05 and power of 80% with a two-sided test, the required number of evaluable patients in both arms was 292, or a total of 321 patients (assuming 10% dropout rate). With the expected accrual of 100 patients per year, the trial was scheduled to close in 4 years.

All analyses were performed according to the ‘intention-to-treat’ principle. Categorical data were analysed with the use of *χ*^2^ test or Fisher's exact test. Continuous data were analysed with Mann–Whitney *U*-test. The mean symptom scores (assessed by patient questionnaires) together with its 95% confidence intervals were plotted to analyse symptomatic improvement with time and according to study group. Survival analysis was performed with Kaplan–Meier method. Survival time was calculated from the date of randomisation until the date of death, survivors being censored at the last date known to be alive. Groups were compared with log-rank test in the univariate analysis. Cox's proportional hazard model was used for multivariate analysis with forward-stepwise regression based on Wald's statistic. Type I error of 0.05 was used for hypothesis testing with no adjustments for multiple comparisons.

## RESULTS

Between September 1997 and April 2000, 100 patients (55 in 20 Gy/5 fr arm and 45 in 16 Gy/2 fr arm) from eight Polish centres were entered into the trial. The trial was closed prematurely due to decreasing accrual. There were 90 men and 10 women aged 47–81 years (median 66), PS 1–4 (median 2). In all, 84 patients had locally advanced tumour and 16 patients additionally had metastatic lesions outside the thorax. Squamous cell carcinoma was diagnosed in 65 patients, adenocarcinoma in nine, large cell carcinoma in one and unspecified NSCLC in 25. There was no significant difference in the distribution of patient characteristics between the two treatment groups (Table 1) and neither was there a major difference in the incidence and degree of initial disease-related symptoms (Table 2).

A total of 98 patients received assigned treatment, whereas two patients died before its completion. Treatment portals did not differ between treatment arms (20 Gy/5 fr: mean 150 cm^2^, range 100–222 cm^2^; 16 Gy/2 fr: mean 146 cm^2^, range 80–218 cm^2^; *P*=0.49).

A total of 58 patients (73% of 80 patients surviving more than 2 months) returned the questionnaire inquiring about their symptoms during the first 8 weeks after the randomisation and were therefore evaluable for symptomatic response. Compliance was similar in both treatment groups (*P*=0.48). The physician assessment of treatment effect was available for 57 patients and radiological assessment of tumour regression for 47 patients.

Treatment tolerance was good and did not differ between the study arms. Side effects in the control and study arms, as reported by treating physicians, included oesophagitis (respectively 12.5 and 24%, *P*=0.30), pneumonitis (respectively 3 and 4%, *P*=1.00), chest pain (respectively 3 and 4%, *P*=1.00) and skin reactions (respectively 3 and 4%, *P*=1.00). There were no detected cases of radiation myelopathy.

**Figure 1:**
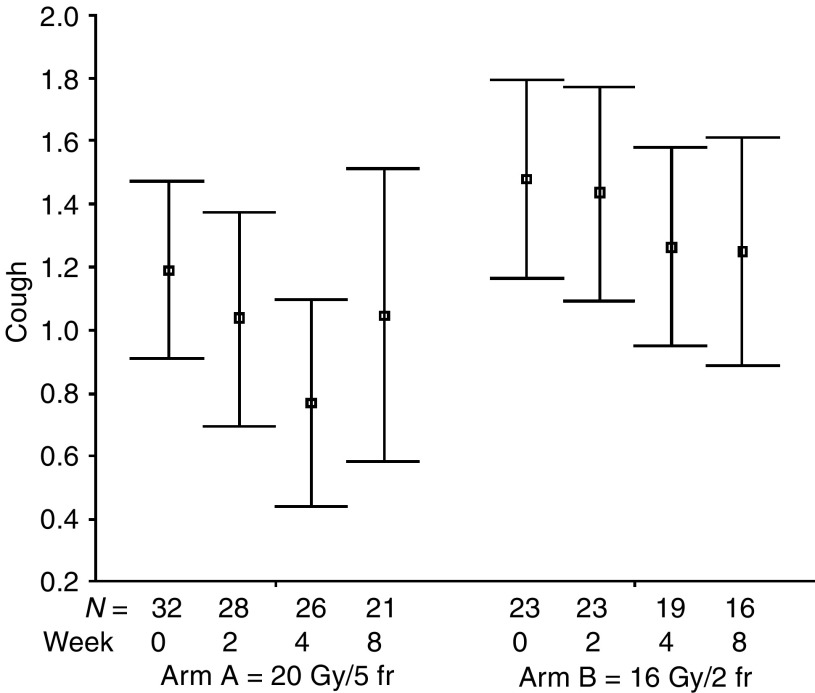
Patients' self-assessment of cough, according to treatment arm and week of follow-up. Boxplots represent mean ±95% confidence interval; *N*=number of patients.

**Figure 2:**
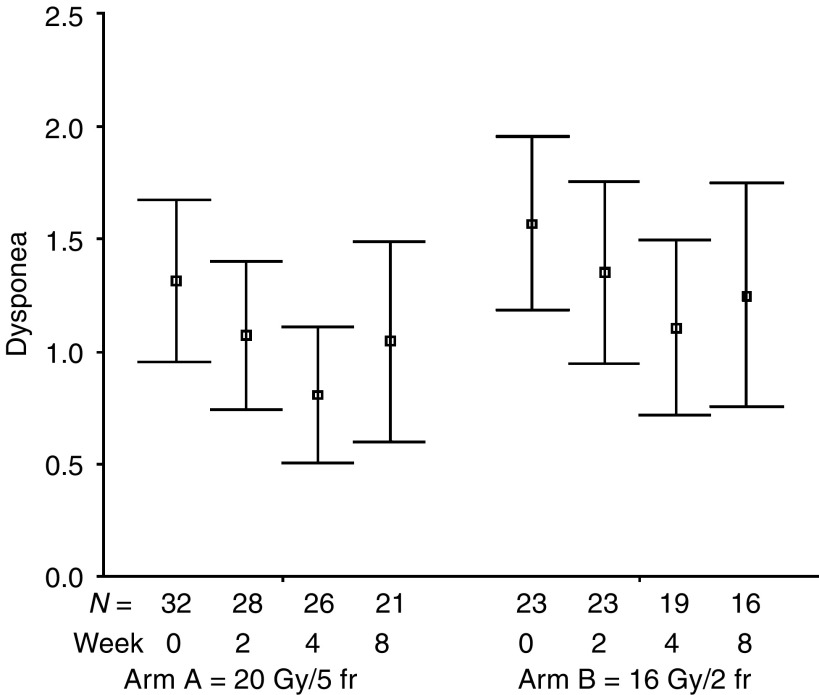
Patients' self-assessment of dyspnoea, according to treatment arm and week of follow-up. Boxplots represent mean ±95% confidence interval; *N*=number of patients.

**Figure 3:**
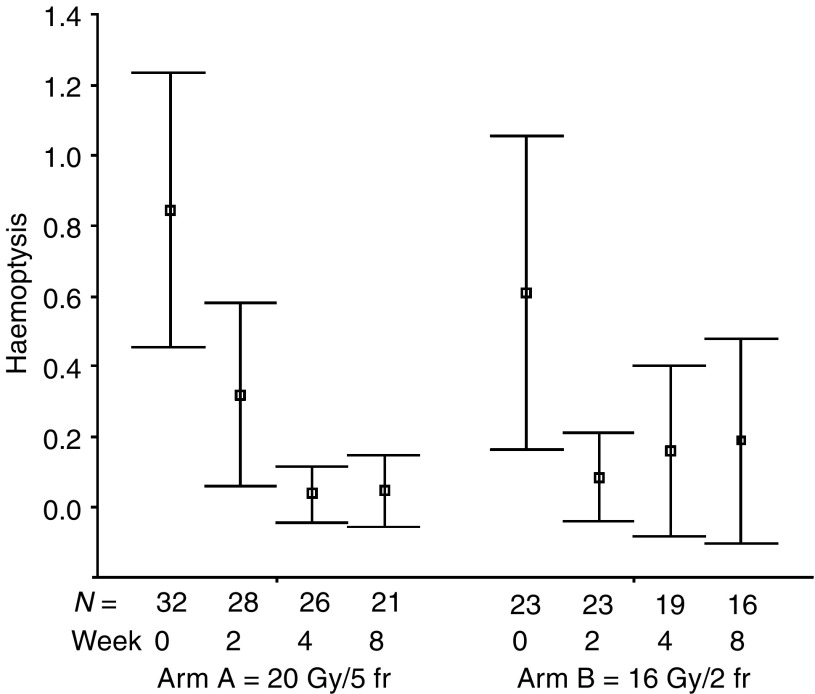
Patients' self-assessment of haemoptysis, according to treatment arm and week of follow-up. Boxplots represent mean ±95% confidence interval; *N*=number of patients.

**Figure 4:**
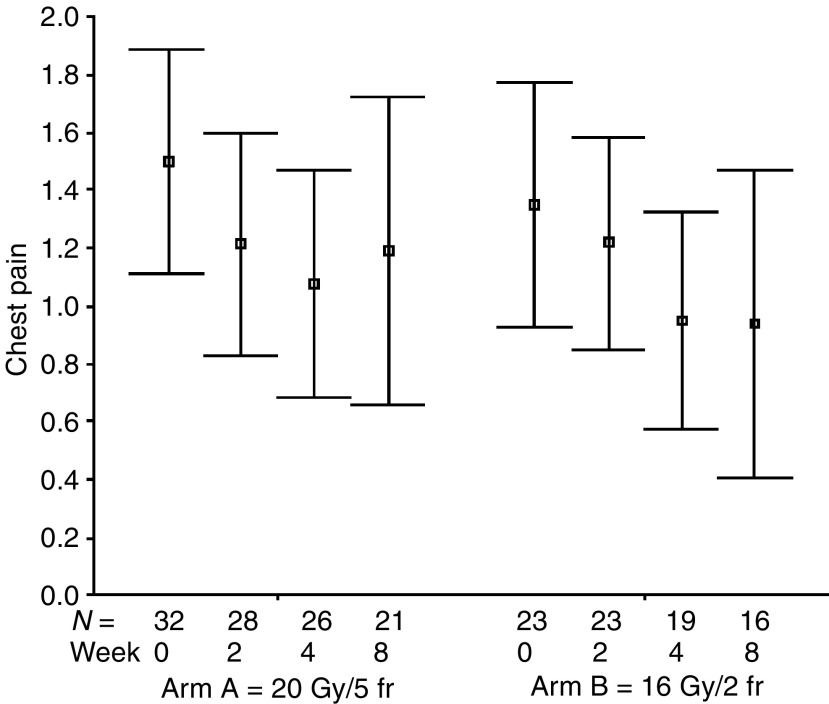
Patients' self-assessment of chest pain, according to treatment arm and week of follow-up. Boxplots represent mean ±95% confidence interval; *N*=number of patients.

**Figure 5:**
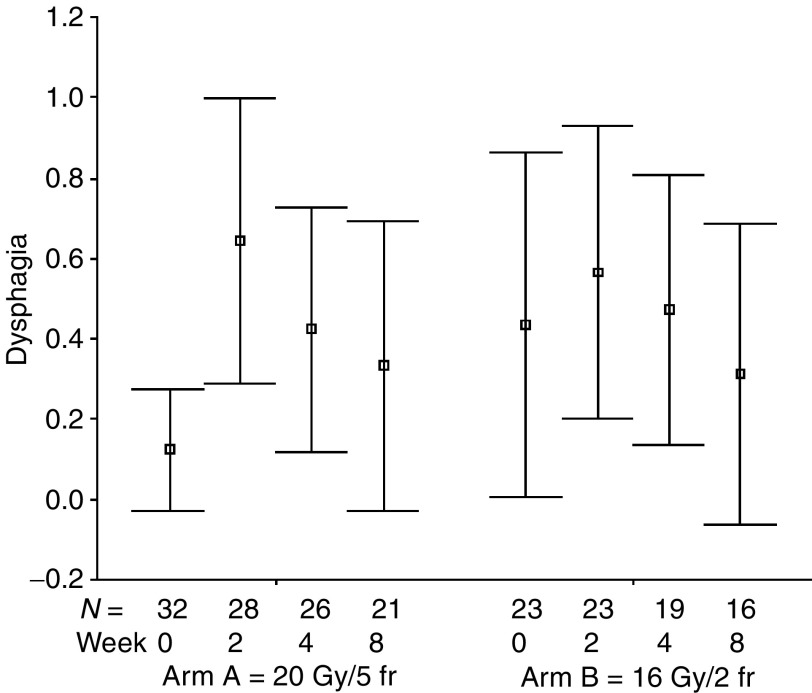
Patients' self-assessment of dysphagia, according to treatment arm and week of follow-up. Boxplots represent mean ±95% confidence interval; *N*=number of patients.

**Figure 6:**
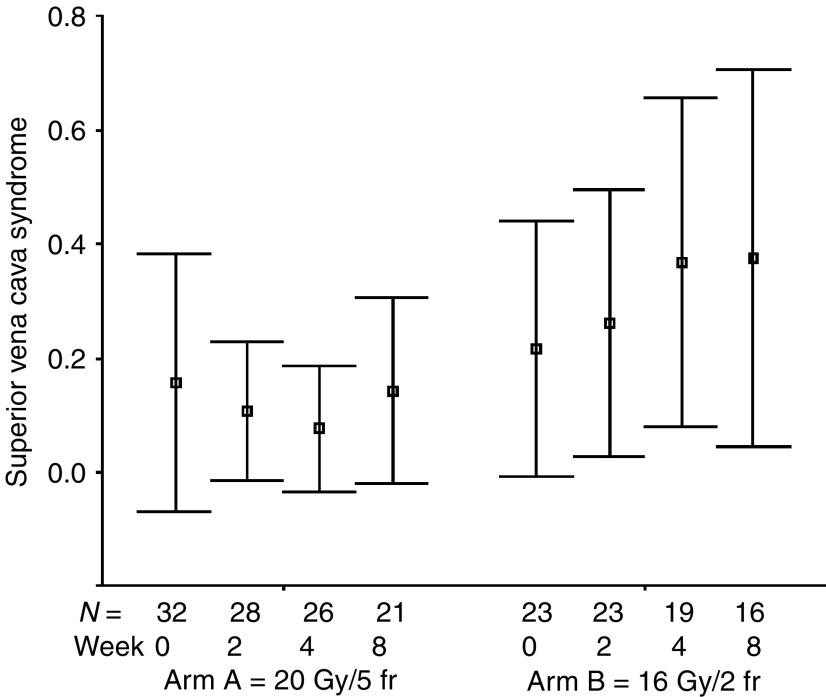
Patients' self-assessment of superior vena cava syndrome, according to treatment arm and week of follow-up. Boxplots represent mean ±95% confidence interval; *N*=number of patients.

The percentages of all evaluable patients reporting any symptomatic improvement were as follows: cough 51% (24 out of 47; for a median of six assessments, range: 1–10), dyspnoea 60% (26 out of 43; for a median of six assessments, range: 1–9), haemoptysis 86% (19 out of 22; for a median of eight assessments, range: 1–11), chest pain 83% (34 out of 41; for a median of four assessments, range: 1–9), dysphagia 71% (five out of seven; for a median of eight assessments, range: 1–9), SVCS 83% (five out of six; for a median of five assessments, range: 4–9). The numbers of patients achieving symptomatic improvement did not differ between study groups for all analysed symptoms (Table 3). The mean symptom scores at specified time intervals after treatment start are presented in Figures 1, 2, 3, 4, 5 and 6. Consistently with percentage of patients reporting symptomatic improvement, the degree of improvement (reduction of symptom score) was highest for haemoptysis and chest pain, and did not differ between study groups, as indicated by overlapping confidence intervals at specific points of time. No difference was also noted between study arms for degree of improvement of dyspnoea, cough and SVCS. Not surprisingly, mean dysphagia scores were higher shortly after radiotherapy in both groups; however, they did not differ according to treatment arm.

No differences in the overall treatment effects as assessed by treating physicians or in the objective response on chest X-ray were observed (Table 4).

During the follow-up period, eight patients (15%) from the 20 Gy/5 fr group and three (7%) from the 16 Gy/2 fr group required additional thoracic radiotherapy after a median of, respectively, 3.5 months (range 3–15 months) and 2 months (range 1–4 months). Three and two patients, respectively, from each arm, were referred for palliative chemotherapy.

**Figure 7:**
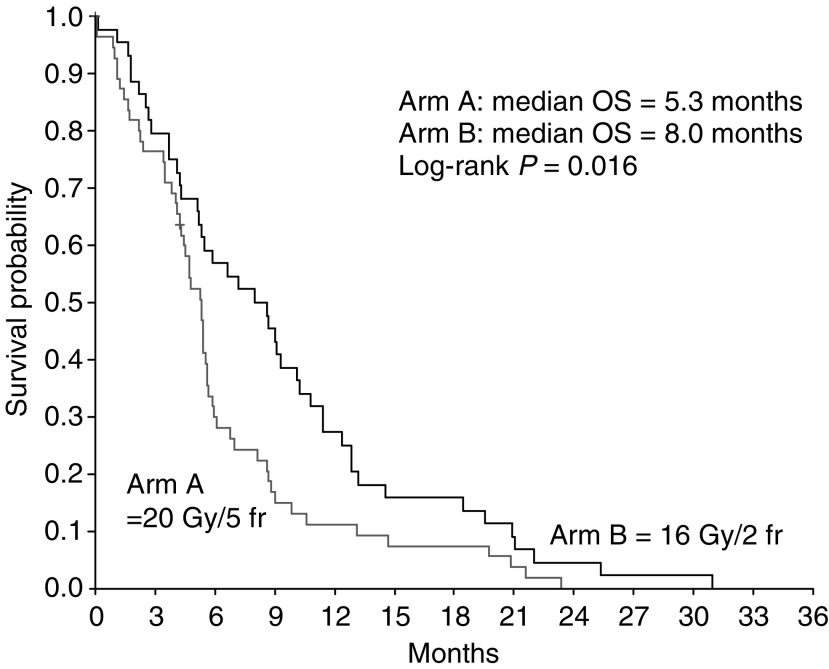
Survival according to treatment group (*n*=100).

Of the 100 patients, 98 have died and two were lost to follow-up. Overall survival time differed significantly between the study groups in favour of 16 Gy/2 fr (median 8.0 months), compared to 20 Gy/5 fr (median 5.3 months), *P*=0.016 (Figure 7). In all, 6- and 12-month survival probabilities were 57% (95% CI: 42–72%) and 27% (95% CI: 14–40%) for patients receiving 16 Gy/2 fr, and 30% (95% CI: 18–42%) and 11% (95% CI: 3–20%) for patients receiving 20 Gy/5 fr, respectively. This difference remained significant after correction for disease extent (*P*=0.022) and performance status (*P*=0.007). Univariate analysis of overall survival according to disease extent, performance status, initial symptoms and radiation treatment is presented in Table 5. Apart from treatment regimen, dysphagia at presentation was the only unfavourable prognostic factor in the univariate analysis (*P*<0.001). In the multivariate analysis, schedule of radiation remained the only prognostic variable, with a hazard ratio of 1.65 (95% CI: 1.09–2.48) for patients administered 20 Gy in 5 fractions as compared to patients who received 16 Gy/2 fr (*P*=0.017).

## DISCUSSION

The issue of optimal palliative irradiation schedule in symptomatic NSCLC has been a subject of numerous prospective randomised studies (Table 6) (Medical Research Council Lung Cancer Working Party, 1991; Medical Research Council Lung Cancer Working Party, 1992; Simpson *et al*, 1985; Teo *et al*, 1988; Abratt *et al*, 1995; Macbeth *et al*, 1996; Rees *et al*, 1997; Reinfuss *et al*, 1999; Nestle *et al*, 2000; Gaze *et al*, 2001; Bezjak *et al*, 2002; Kramer *et al*, 2003; Sundstrom *et al*, 2004). Although the comparison of these trials is difficult due to differences in the radiotherapy regimens, patient characteristics and outcome measures, there is no strong evidence for the superiority of any particular regimen (Hansen, 2002; Macbeth *et al*, 2004). Probably the most important and influential trials were those conducted consecutively in the UK by the Medical Research Council (MRC). These studies were first to demonstrate the feasibility and efficacy of very short radiotherapy regimens of two fractions of 8.5 Gy (Medical Research Council Lung Cancer Working Party, 1991) or one fraction of 10 Gy (Medical Research Council Lung Cancer Working Party, 1992). The results of these studies were generally confirmed by subsequent trials (Simpson *et al*, 1985; Abratt *et al*, 1995; Macbeth *et al*, 1996; Rees *et al*, 1997; Nestle *et al*, 2000; Kramer *et al*, 2003; Sundstrom *et al*, 2004) and are in agreement with the results of our study. Importantly, like all these studies, we used relatively simple treatment planning system rather than sophisticated three-dimensional methods used in protracted radiotherapy regimens. Indeed, these easy to administer and nontoxic regimens resulted in effective and durable palliation of main symptoms (Medical Research Council Lung Cancer Working Party, 1991; Medical Research Council Lung Cancer Working Party, 1992; Sundstrom *et al*, 2004). These results, however, were challenged by a few studies, which demonstrated better palliation in patients given higher radiation doses (Teo *et al*, 1988; Gaze *et al*, 2001; Bezjak *et al*, 2002). These discrepancies can at least partially be explained by various end points and differences in evaluation tools used in particular studies (Bezjak *et al*, 2002). In particular, many studies emphasised the importance of relying (as we did) more on patient self-assessment than on physicians' evaluation, as major differences are observed between results of both these judgments (Macbeth *et al*, 1996; Stout *et al*, 2000; Sundstrom *et al*, 2004).

The major concern related to the use of hypofractionated treatment schedules is their potential inferiority in terms of overall survival (Macbeth *et al*, 1996; Bezjak *et al*, 2002). Some evidence exists that higher radiotherapy doses result in a modest increase in survival, although at the expense of higher acute toxicity (Macbeth *et al*, 1996; Reinfuss *et al*, 1999; Bezjak *et al*, 2002; Kramer *et al*, 2003; Macbeth *et al*, 2004; Sundstrom *et al*, 2004). The effect of radiotherapy dose and regimen on overall survival, if any, was in all instances limited to patients with good PS and/or relatively nonadvanced disease, that is, those most likely to benefit from improved local control (Kowalska, 1992; Macbeth *et al*, 1996; Reinfuss *et al*, 1999; Bezjak *et al*, 2002; Sundstrom *et al*, 2004). In contrast to these results, our study demonstrated improved survival in the shorter treatment arm and this difference remained significant after correction for disease extent and performance status. This intriguing result should, however, be interpreted with caution due to relatively small number of patients in the study arms. Performance status and disease stage, of confirmed prognostic value in other studies (Medical Research Council Lung Cancer Working Party, 1991; Kowalska, 1992), had no independent impact on overall survival in our series. It is possible that the survival difference in this study was associated with some undiscovered imbalance between treatment groups due to small sample size caused by premature termination of the study and its simple and pragmatic design (e.g., no detailed TNM staging was requested). The imbalance in the number of PS 3 or 4 patients between both study groups, although not statistically significant by Pearson's *χ*^2^ test (with small numbers in subgroups), might have potentially translated into survival difference. As mentioned, PS did not, however, affect survival in univariate or multivariate analysis. It would be difficult to find another explanation for improved survival in patients receiving less treatment, although in two other studies a trend toward improved survival in the lower dose group was observed in a subset analysis (Nestle *et al*, 2000; Sundstrom *et al*, 2004). It seems reassuring that such a short treatment is at least not inferior in terms of survival, compared to a standard schedule.

As a result of published trials, a general conclusion was made (and became a basis for official recommendations) that selected advanced and symptomatic NSCLC patients should be treated with just 1 or 2 fractions of palliative radiotherapy (American Society of Clinical Oncology, 1997; Jassem, 2001; Macbeth *et al*, 2004). Such a policy has been widely adopted in the United Kingdom (Priestman, 1996), but not in many other parts of the world (Bezjak *et al*, 2002). Apart from purely medical factors, such an approach has obvious logistic and economical benefits, which is of particular importance in countries with limited health care resources. Commonly used treatment schedules are still, however, more often based on tradition than on clinical research results (Macbeth *et al*, 2004). In particular countries treatment policy is a subject of different societal, cultural, attitudinal and health service delivery influences (Bezjak *et al*, 2002). The sources of reluctance toward hypofractionated regimens include the lack of experience with large single fraction, concerns about its acute toxicity and uncertainty about the appropriate patient selection for hypofractionated therapy (Bezjak *et al*, 2002). The main rationale for the use of larger radiotherapy doses and longer fractionation schemes, apart from potential survival gain, is improvement in local control leading to better quality of life (Macbeth *et al*, 1996). Indeed, in some studies during long follow-up, better palliative effect was observed in patients applied protracted schedules (Macbeth *et al*, 1996). On the other hand, short regimens allow for more rapid symptom control (Macbeth *et al*, 1996). As one of the aims of palliative radiotherapy is psychological support, another worry related to the use of very short fractionation regimens is their potentially negative effect on patients' psychological functions, such as levels of anxiety or depression (Falk *et al*, 2002). In one study, increased anxiety was observed in patients treated with one fraction, compared to those administered 10 fractions (Gaze *et al*, 2001). No negative effect on anxiety, depression or psychological distress was seen, however, in patients assigned to delayed rather than immediate radiotherapy in the MRC study (Falk *et al*, 2002). These differences could possibly have been caused by varying use of other, complementary methods of psychological support (Falk *et al*, 2002).

The efficacy of palliative radiotherapy depends on the type of predominant symptom. Several studies, including the present one, demonstrated that the most effectively palliated symptoms include haemoptysis and chest pain (Simpson *et al*, 1985; Macbeth *et al*, 1996; Rees *et al*, 1997; Cross *et al*, 2004; Sundstrom *et al*, 2004). In some studies, irradiation also resulted in effective relief of cough – the symptom least effectively palliated in our series, as well as in the recently reported Norwegian study (Rees *et al*, 1997; Sundstrom *et al*, 2004). It is of note that radiotherapy was also found to relieve effectively general symptoms (not directly related to chest tumour), like lack of energy, tiredness or anorexia (Medical Research Council Lung Cancer Working Party, 1991; Macbeth *et al*, 1996; Cross *et al*, 2004). This treatment modality seems to be least effective for dyspnoea, which in some patients may be related to irreversible lung damage caused by pulmonary collapse or consolidation (Simpson *et al*, 1985; Stout *et al*, 2000; Langendijk *et al*, 2001; Cross *et al*, 2004).

The modern definition of palliation (as recommended by the MRC Cancer Trials Office) encompasses symptom improvement (reduction of existing moderate or severe symptoms), control (no deterioration in mild symptoms) and prevention (no deterioration in those with no symptoms) (Stephens *et al*, 1999). Such a comprehensive assessment is particularly important in the setting of lung cancer, a tumour typically accompanied by multiple symptoms (Bezjak *et al*, 2002). Our study was initiated before these recommendations were published and was designed to measure predominantly the degree of symptomatic improvement. Nevertheless (although not planned in the study protocol), the evaluation of the mean score of symptom intensity encompassed also the development of new symptoms and allowed for some estimate of the efficacy of compared treatments in their control and prevention.

An unanswered question remains the optimal management of asymptomatic or minimally symptomatic NSCLC patients not suitable for radical treatment, in whom one of the options is watchful waiting. The argument for early treatment in these patients is that enhanced local control may prolong survival and improve quality of life by delaying development of thoracic symptoms (Falk *et al*, 2002). The results of randomised studies testing early *vs* delayed or ‘as required’ radiotherapy in this group of patients are contradictory (Roswit *et al*, 1968; Durrant *et al*, 1971; Reinfuss *et al*, 1999; Falk *et al*, 2002). A Polish study demonstrated a major difference in overall survival in favour of early treatment (Reinfuss *et al*, 1999), whereas in the recent MRC trial no differences in main outcome measures, including overall survival, were observed (except for a delay in the development of severe or moderate symptoms in the early treatment group) (Falk *et al*, 2002). Importantly, 58% of patients in the ‘delayed treatment’ group never needed thoracic radiotherapy (Falk *et al*, 2002; Hansen, 2002). The observed discrepancies in patient survival may result from patient selection or differences in radiotherapy regimen used (relatively high doses in the Polish study *vs* 1–2 fractions in the MRC study). It is also possible that patients in early irradiation groups might possibly have been offered better supervision and supportive care (Roswit *et al*, 1968; Reinfuss *et al*, 1999; Falk *et al*, 2002).

Our trial was closed prematurely due to decreasing accrual of patients. As in other similar studies, this poor outcome might partially be due to the increasing application of palliative chemotherapy, which has recently come into widespread use in this population of patients (Langendijk *et al*, 2001; Cross *et al*, 2004). The limited number of patients accrued into the study obviously decreases its statistical power to detect potential differences in outcome. The early closure of the study also resulted in the unequal numbers of patients in both arms. Indeed, the design of randomisation method (randomisation in blocks after stratification) was created to assure balance between originally planned, larger groups of patients. A number patients in this series was diagnosed using fine-needle aspiration biopsy of primary tumour or peripheral lymph nodes, thus creating a relatively large proportion of unclassified NSCLCs. In fact, no attempt was made to specify the diagnosis further, as this would have had no therapeutic implications. Another problem encountered in our study was poor patient compliance in completing questionnaires and attending follow-up visits. This problem, observed also in other studies, can partially be related to patient selection, as those surviving less than 3 months are unlikely to comply with the study requirements. These patients also usually do not benefit from radiotherapy, therefore their inclusion may ‘dilute’ real treatment outcomes (Medical Research Council Lung Cancer Working Party, 1992; Macbeth *et al*, 1996; Rees *et al*, 1997). Furthermore, obviously not all symptoms were present in all patients, making statistical analysis more difficult (Bezjak *et al*, 2002). In future studies, this problems can perhaps be overcome by the assessment of ‘index symptom’, that is, the single most troublesome symptom in each patient, constituting the primary indication for palliative radiotherapy (Bezjak *et al*, 2002). It may also be valuable to derive some aggregated variable lumping scores of key symptoms. In the current study, however, no difference was observed between treatment arms in the degree of symptomatic improvement after radiotherapy, therefore no further, derivate variables assessing the effect of radiotherapy were analysed.

Our study was carried out between 1997 and 2000. Importantly, for many of these patients current standard of treatment would include chemotherapy, which was demonstrated to be effective both as a sole modality in the palliative setting, and in combination with radiotherapy in locally advanced NSCLC. The majority of patients included in current study were not, however, candidates for combined modality approaches, in particular to curative therapy (as clearly specified by the inclusion criteria). Some patients actually received palliative chemotherapy at some time during the course of their disease, but this could not substantially influence the main study results.

To conclude, our study confirmed the equal efficacy of shorter *vs* longer palliative lung cancer radiotherapy schedules in terms of palliative effect and treatment tolerance. An improvement in overall survival was observed in patients treated with 16 Gy/2 fr, confirming the efficacy of this approach. This may hopefully convince at least some radiation oncologists still using more protracted regimens to adopt this simple and efficient treatment.

## Figures and Tables

**Table 1:** Patient characteristics at randomisation

**Patient's characteristics**	**20 Gy/5 fr**	**16 Gy/2 fr**	***P***
*Gender*			
Male	48	42	0.51
Female	7	3	
			
*Age (years)*			
Median	67	66	0.73
Range	47–81	52–79	
			
*Disease extent*			
Locally advanced	45	39	0.70
Metastatic	10	6	
			
*Pathology*			
Squamous cell	35	30	0.62
Adenocarcinoma	6	3	
Large cell	—	1	
Unspecified NSCLC	14	11	
			
*WHO performance status*			
PS 1	17	22	0.16
PS 2	27	18	
PS 3	8	5	
PS 4	3	—	

**Table 2:** Initial symptoms of chest tumour

**Symptom**	**20 Gy/5 fr**	**16 Gy/2 fr**	***P***
*Cough*			
Mild	15	14	0.65
Moderate	14	16	
Severe	2	1	
			
*Dyspnoea*			
Mild	10	9	0.32
Moderate	13	17	
Severe	9	3	
			
*Haemoptysis*			
Mild	9	6	0.62
Moderate	9	5	
Severe	1	2	
			
*Chest pain*			
Mild	8	13	0.44
Moderate	17	11	
Severe	7	5	
			
*Dysphagia*			
Mild	5	1	0.14
Moderate	1	—	
Severe	—	2	
			
*SVCS*			
Mild	2	2	0.82
Moderate	1	1	
Severe	1	—	

**Table 3:** Numbers of patients reporting symptomatic improvement by treatment group

**Symptom**	**20 Gy/5 fr**		**16 Gy/2 fr**		
	**Number** **improving**	**Median number of** **assessments with** **improvement (range)**	**Number** **improving**	**Median number of** **assessments with** **improvement (range)**	
Cough	12/26 (46%)	6 (1–8)	12/21 (57%)	6 (1–10)	0.45
Dyspnoea	13/23 (54%)	4 (1–8)	13/20 (65%)	6 (1–9)	0.57
Haemoptysis	12/15 (80%)	7 (1–10)	7/7 (100%)	8 (1–11)	0.52
Chest pain	20/24 (83%)	4 (1–9)	14/17 (82%)	4 (1–8)	1.00
Dysphagia	2/3 (67%)	3 (1–4)	3/4 (75%)	8 (8–9)	1.00
SVSC	2/2 (100%)	7 (5–9)	3/4 (75%)	5 (4–7)	1.00

*Note*: denominators of numbers of patients improving in each group may differ from data included in Table 2, as only patients with available follow-up were included in the analysis.

**Table 4:** Physician and radiologic assessment of therapeutic effect by treatment group

	**20 Gy/5 fr**	**16 Gy/2 fr**	***P***
*Physician assessment*			
Complete symptomatic response	6 (19%)	4 (16%)	0.69
Improvement	17 (53%)	15 (60%)	
No change	5 (16%)	5 (20%)	
Worsening of symptoms	4 (12%)	1 (4%)	
			
*Radiologic assessment*			
Complete response	—	—	0.99
Partial response	12 (52%)	13 (54%)	
No change	7 (31%)	7 (29%)	
Progression	4 (17%)	4 (17%)	

**Table 5:** Univariate analysis of overall survival (OS)

	***N***	**Median OS (months)**	**95% CI**	***P* (log-rank)**
*Disease extent*				
Locally advanced	84	5.6	(5.0–6.2)	0.115
Metastatic	16	4.2	(0.8–7.6)	
				
*Performance status*				
1	39	5.5	(5.2–5.8)	0.091
2	45	5.7	(3.7–7.6)	
>2	16	3.8	(1.5–6.2)	
				
*Cough*				
No	17	6.0	(5.2–6.7)	0.586
Yes	62	5.3	(4.4–6.1)	
				
*Dyspnoea*				
No	18	5.6	(4.6–6.5)	0.624
Yes	61	5.4	(4.4–6.4)	
				
*Haemoptysis*				
No	47	5.5	(5.1–5.8)	0.738
Yes	32	5.4	(1.1–9.7)	
				
*Chest pain*				
No	18	4.8	(2.5–7.0)	0.333
Yes	61	5.6	(4.9–6.3)	
				
*Dysphagia*				
No	70	5.9	(4.7–7.0)	<0.001
Yes	9	4.3	(0.0–9.7)	
				
*SVCS*				
No	72	5.4	(4.8–6.1)	0.365
Yes	7	5.6	(2.0–8.3)	
				
*Treatment arm*				
20 Gy/5 fr	55	5.3	(4.6–6.0)	0.016
16 Gy/2 fr	45	8.0	(4.5–11.4)	

**Table 6:** Randomised studies of palliative lung cancer radiotherapy

**Reference**	**Treatment schedules**	**Entry criteria**	**Treatment tolerance**	**Palliative effect**	**Overall survival**
MRC I (1)	30 Gy/10 × *vs* 17 Gy/2 ×	Locally advanced NSCLC (including M+)	No difference	No difference	No difference
MRC II (2)	17 Gy/2 × *vs* 10 Gy/1 ×	PS⩾2, inoperable NSCLC (including M+)	More dysphagia with 17 Gy/2 ×	No difference	No difference
Rees (3)	22.5 Gy/5 × *vs* 17 Gy/2 ×	Lung cancer suitable for palliative chest RT	Trend for more dysphagia with 17 Gy/2 ×	Trend for better control of chest pain and cough in 17 Gy/2 ×	No difference
MRC (4)	39 Gy/13 × *vs* 17 Gy/2 ×	Locally advanced NSCLC, PS 0–2	More dysphagia with 39 Gy/13 ×	More rapid palliation in 17 Gy/ 2 ×	Improved OS in 39 Gy/13 ×
RTOG (5)	40 Gy/10 × (split course) *vs* 30 Gy/10 × *vs* 40 Gy/20 ×	Locally advanced NSCLC, Karnofsky ⩾60	More pneumonitis with 40 Gy/ 10 × (split course)	No difference	No difference
Reinfuss (6)	50 Gy/25 × *vs* 40 Gy/10 × (split course) *vs* observation	Stage III, unresectable, asymptomatic NSCLC	More grade 2 oesophagitis with 40 Gy/10 × (split course)	NA	Improved OS in 50 Gy/25 ×
Nestle (7)	60 Gy/30 × *vs* 32 Gy/16 × (bid)	Inoperable NSCLC (stage III or ‘minimal’ IV), Karnofsky ⩾50	Earlier oesophagitis with 32 Gy/ 16 × (bid)	No difference	No difference
Abratt (8)	45 Gy/15 × *vs* 35 Gy/10 ×	Stage III NSCLC not suitable for radical RT	More oesophagitis with 45 Gy/ 15 ×	No difference	No difference
Teo (9)	45 Gy/18 × *vs* 31.2 Gy/4 ×	Inoperable NSCLC not suitable for radical RT (including M+)	No difference	Better palliation in 45 Gy/18 ×	No difference
NCIC CTG SC.15 (10)	20 Gy/5 × *vs* 10 Gy/1 ×	Locally advanced NSCLC (including M+)	No difference	Better palliation in 20 Gy/5 ×	Improved OS in 20 Gy/5 ×
Gaze (11)	30 Gy/10 × *vs* 10 Gy/1 ×	Advanced NSCLC	Not reported	Better palliation in 30 Gy/10 ×	No difference
Sundstrom [Z]	50 Gy/25 × *vs* 42 Gy/15 × *vs* 17 Gy/2 ×	Locally advanced NSCLC (including M+)	Less and later occurrence of dysphagia with 50 Gy/25 arm	No difference	No difference
Dutch [Y]	30 Gy/10 × *vs* 16 Gy/2 ×	Locally advanced NSCLC (including M+)	More complains in 16 Gy/2 ×	Earlier response in 16 Gy/2 ×	Improved OS in 30 Gy/10 ×
Current study	20 Gy/5 × *vs* 16 Gy/2 ×	Locally advanced NSCLC (including M+)	No difference	No difference	Improved OS in 16 Gy/2 ×

M+=metastatic, MRC=Medical Research Council, NA=not applicable, NCIC=National Cancer Institute of Canada, NSCLC=non-small-cell lung cancer, OS=overall survival, PS=performance status, RT=radiotherapy, RTOG=Radiation Therapy and Oncology Group.
